# Making and Receiving Offers of Help on Social Media Following Disaster Predict Posttraumatic Growth but not Posttraumatic Stress

**DOI:** 10.1017/dmp.2020.43

**Published:** 2021-08

**Authors:** Yael Levaot, Talya Greene, Yuval Palgi

**Affiliations:** Department of Community Mental Health, University of Haifa, Mount Carmel, Israel; Department of Gerontology and the Center for Research and Study of Aging, University of Haifa, Mount Carmel, Israel

**Keywords:** fire, posttraumatic growth, posttraumatic stress, social media, trauma

## Abstract

**Objectives:**

Social media provides an opportunity to engage in social contact and to give and receive help by means of online social networks. Social support following trauma exposure, even in a virtual community, may reduce feelings of helplessness and isolation, and, therefore, reduce posttraumatic stress symptoms (PTS), and increase posttraumatic growth (PTG). The current study aimed to assess whether giving and/or receiving offers of help by means of social media following large community fires predicted PTS and/or PTG.

**Methods:**

A convenience sample of 212 adults living in communities that were affected by large-scale community fires in Israel (November 2016) completed questionnaires on giving and receiving offers of help by means of social media within 1 mo of the fire (W1), and the PTSD checklist for DSM-5 (PCL-5) and PTG questionnaire (PTGI-SF), 4 mo after the fire (W2).

**Results:**

Regression analyses showed that, after controlling for age, gender, and distance from fire, offering help by means of social media predicted higher PTG (*β* = 0.22; *t* = 3.18; *P* < 0.01), as did receiving offers of help by means of social media (*β* = 0.18; *t* = 2.64; *P* < 0.01). There were no significant associations between giving and/or receiving offers of help and PTS.

**Conclusions:**

Connecting people to social media networks may help in promoting posttraumatic growth, although might not impact on posttraumatic symptoms. This is one of the first studies to highlight empirically the advantages of social media in the aftermath of trauma exposure.

Studies indicate that exposure to a natural or man-made disaster is associated with elevated rates of posttraumatic stress disorder (PTSD), depression, and other anxiety disorders.^1^ In line with this, it has been shown that mental health may be adversely affected following exposure to fires in Australia,^2,3^ California,^4^ and Israel.^5^ On the other hand, research has indicated that not all outcomes following trauma exposure are necessarily adverse, and that people may also experience positive outcomes.^6^

Posttraumatic growth (PTG) is defined as positive psychological change experienced as a result of the struggle with a difficult life circumstance.^6^ A traumatic event may challenge core beliefs, forcing reassessment of belief systems, and, therefore, might enhance PTG.^7^ The relationship between posttraumatic stress symptoms (PTS) and PTG is unclear.^8^ Levine et al.^9^ found curvilinear associations between the two, with the highest levels of PTG among those with moderate levels of PTS, while others found that PTG is prevalent among those who screen positive for PTS and showed positive linear associations between PTS and PTG.^10,11^ In a meta-analysis, Shakespeare-Finch and Lurie-Beck^12^ found a significant linear relationship between PTG and PTS symptoms but also a significantly stronger curvilinear relationship.

A significant body of literature suggests that postevent factors play a critical role in influencing mental health.^2,13-17^ Both PTS and PTG are related to the fact that individuals live in an environment in which traumatic events and other factors interact, with the progressive accumulation of risk and resilience.^15,18^ These factors include subsequent events, resources, community functioning, social support, and others. Together, they can compound or buffer the mental health reactions following trauma.

Exposure to traumatic events can occur through experiencing a trauma firsthand or witnessing a trauma as it occurs to others, or through secondary narrative accounts (APA, 2013), and even through media reports that have been shown to be associated with PTSD.^19^ Yeung et al.^20^ found that, among people who were not directly exposed to a traumatic event, exposure to distressing media images, emotional responses, and disaster-related perceptions, were predictive of probable PTSD several months later.

Social media is becoming increasingly important in both daily life and following disasters.^21^ This growth in social media use may increase the likelihood of indirect trauma exposure through the sharing and viewing of uncensored pictures, videos, and other content.^22,23^ Along with these risks, social media provide benefits to users. Compared with face-to-face encounters, social media users can choose their own level of engagement, and as a result experience greater control.^24^ Furthermore, online communities might be suitable for receiving social support after traumatic events, regardless of geographical location or time.^25^ Perceived social support is a significant predictor of PTG^26^ and can be used to enhance mental health.^27^ Social support, even in a virtual community, may provide a way of improving feelings of helplessness and reducing isolation through exchange in an online messages.

Relatively little research has focused on motivations for social support provided through social media.^28^ Lee et al.^29^ found that new media use correlated positively with various dimensions of social support and revealed that positive social interaction, and emotional and informational social support were the strongest determinants of quality of life regardless of whether it came from online or offline sources. Baker and Yang^30^ argued that a widely accepted source of social support today is by means of social media platforms. Therefore, social media might function as a central component in coping with the aftermath of a disaster and provide an opportunity to engage in social contact through giving and receiving help by joining online social networks and discussions.^31^

There are 2 key types of social media use: active and passive.^32^ Active social media use has been described as the production of content and interaction between a user and another friend.^33^ Active social media use might also include posting comments or “likes” to content with which the user has no direct relationship. It can be described as a way of identity expression, both self and social.^34^ Active social media use has been shown to affect users beneficially and reduce loneliness and depression.^35^ Previous research has noted the advantage of online narrating and interaction, which provides the benefit of both anonymity and self-disclosure.^36^

In the context of trauma exposure, the use of video recordings, blogs, and forum postings has been described as a fast and simple way to share authentic narratives and to communicate.^37^ Research to date has been uncertain on determining how media use of any type after traumatic events elevates or regulates anxiety and distress.^38^ In a qualitative analysis of narratives, Salzmann-Erikson and Hicdurmaz^37^ indicate that active social media use provides PTSD sufferers a way to experience the universality of the problem and to receive support from others. Moreover, Yoshida et al.^39^ suggested that viewing media coverage can facilitate deliberate rumination, which can be beneficial for posttraumatic recovery and nurturing PTG, and it may be that this is relevant in the case of social media usage too. However, it is unclear whether social media use following traumatic events is associated with PTG.

The present study examined whether making or receiving offers of help by means of social media following fires predicted PTS and/or PTG in a longitudinal study of adults who were living in an area that was exposed to large community fires in Israel. This study examined the impact of active social media use and specifically investigated whether this use was associated with both PTG and PTS. We hypothesized that both receiving more offers of help and offering help by means of social media would predict lower levels of PTS and higher levels of PTG.

## METHODS

### Participants, Sampling, and Procedure

In November 2016, several large fires erupted throughout Israel, affecting multiple communities. These fires spread rapidly, damaged many properties, and threatened the lives of citizens. According to estimates, 133 people were injured in the fires, around 1800 homes were damaged, and some 70,000 people were temporarily evacuated from their homes (https://www.ynet.co.il/articles/0,7340,L-4884631,00.html).

Data were collected in 2 waves from a convenience sample of individuals living in communities affected by the fires, who were recruited by means of social networking sites and apps, university mailing lists, street outreach, and snowballing methods also described in Palgi et al.^40^ The first wave took place within 1 mo of the fire (December 13, 2016 to December 31, 2016; W1). The sample included 445 individuals aged 18-95 years (*M* = 40.23 SD = 14.33). Most participants completed an online survey using the Qualtrics platform (*n* = 350). Participants recruited by means of street outreach could choose to complete the online survey or complete it by paper and pencil administration (*n* = 22).

To capture symptoms that were after the immediate phase, but would allow us to identify those who developed PTS symptoms or positive change of PTG before 6 mo when the “delayed presentation” of PTS appear (APA, 2013), the second wave took place 4 mo after the fire (March 23, 2017 to April 18, 2017; W2), 212 of the original participants completed online follow-up questionnaires (58.24%) and were included as the study sample. The questionnaire included an informed consent checkbox. Both waves of the study were approved by the Ethics Committee of the University of Haifa.

Of the participants, 73.9% were female (*n* = 176), 63.6% were married (*n* = 135), and most of them had higher education (81.7%; *n* = 173). Participants included those whose homes were destroyed or damaged by the fire (21.7%; *n* = 94), and people who were themselves physically close to the fire (52.6% were in 0- to 500-meter physical distance to the fire).

### Measures

#### Social Media Offers of Help

One item was used to examine social media offers of help; participants were asked “Did you offer help by means of social media?” The response format was a Likert scale that ranged from 0 = not at all, to 4 = to a large extent.

#### Social Media Receiving Help

Participants were asked whether they had received offers of help by means of social media with 3 questions: (1) “Have you received concrete offers of help from people following the fire through social media?” (2) “Have you received emotional offers of help from people following the fire through social media?” (3) “Have you received informative offers of help from people following the fire through social media?” The response format was a Likert scale that ranged from 0 = not at all, to 4 = to a large extent. The total score was the sum of responses across all items.

#### Social Media Use

Two items were used to examine participants’ social media use during and 1 wk after the fire and its perceptions as helpful/stressful. One question assessed the frequency of social media use following the fire: (1) “I did not use social media”; (2) “I used social media less than usual”; (3) “I used social media as usual”; (4) “I used social media more than usual”.

The second question asked participants to indicate whether social media use was experienced as helpful or stressful. Range from 1 = “social media use did not help me and stressed me a lot” to 5 = “social media use helped me a lot”.

#### PTS

PTS were assessed at W2, 4-5 mo after the fire with a translated and back-translated Hebrew version of the PTSD Checklist for DSM-5 (PCL-5, Weathers et al., 2013).^41^ The questionnaire was translated and back-translated. This questionnaire is a 20-item measure and participants rate each symptom experienced during the previous month on a 5-point Likert scale from 0 = not at all to 4 = extremely. Participants were asked to rate each symptom while thinking of the most stressful event related to the fire that they had reported. PTSD symptom score was the sum of ratings. Internal reliability was excellent (Cronbach’s alpha *α* = .95).

#### PTG

PTG was assessed at W2 by a Hebrew version of the Posttraumatic Growth Inventory-Short Form (PTGI-SF, Cann et al., 2010).^42^ The questionnaire was translated and back-translated. Participants were asked to rate each item on a scale that ranged from 0 = “I did not experience this change,” to 4 = “I experienced this change to a great deal.” A sum score was computed across all items. Higher scores indicated higher levels of PTG symptoms. Reliability was excellent (Cronbach’s alpha *α* = .90).

Covariates included the following demographics: age, gender, education and marital status (1 = married or living with a partner; 2 = single; 3 = single-parent, divorced or widowed). In addition, we asked participants: (1) whether they evacuated their homes on the day of the fire, (2) their nearest personal distance to the fires in meters according to the following categories: a = 0-200; b = 200-500; c = 500-1000; d = 1000-2500; e = 2500-5000; f = >5000, (3) the proximity of their home to the fires in meters on the following categories: a = 0-200; b = 200-500; c = 500-1000; d = 1000-2500; e = 2500-5000; f = >5000.

### Data Analysis

Analysis was conducted using SPSS versus 23 software. Linear regression analyses was performed to investigate whether making and/or receiving offers of help by means of social media predicted subsequent PTS and PTG, above and beyond background variables. Covariates (age, gender, marital status, education, and exposure to the fire) were entered in the first step of the regression.

## RESULTS

The demographic characteristics of 212 participants who completed both waves of data collection are summarized in Table 1. Of the participants, 72.4%; (*n* =154) indicated their use of social media outlets following the fires as “more than usual”. Moreover, the majority of participants indicated that social media use was more helpful than stressful on a scale of 0-5 (*M* = 3.82; SD = 1.298). Women reported significantly higher PTG (*M* = 6.28; SE = .592) than men (*M* = 3.15; SE = .679), *t*(198) = 2.578; *P* < 0.01. There were no significant differences on PTS between women and men *t*(198) = 1.344, *P* > 0.05.

**TABLE 1:** Descriptive Statistics and Correlations for the Study Variables

	M/ %	SD	1	2	3	4	5	6	7	8	9
Age	40.23	14.33	-								
Female gender^a^	73.9%										
Currently married or living with a partner^b^	63.9%										
Higher education	81.74%										
Previous types of trauma exposures	3.16	2.61	.139*	.114	.013	−.024					
Personal distance from fire	1.71	1.78	−.04	.059	.091	−.02	−.073				
Receiving offers of help on social media	2.11	1.061	.042	−.130*	−.004	.021	−.025	−.199**			
Making offers of help on social media	2.47	.916	.173**	−.054	.048	.123	.136*	−.063	.352**		
PTG (W2)	5.64	7.045	−.122	−.144*	−.079	−.117	.171*	−.173*	.310**	.287**	
10. PTS (W2)	1.15	1.448	−.216**	−.081	−.297**	−.203**	.291**	−.019	.050	.049	.331**

Abbreviations: PTG, posttraumatic growth; PTS, posttraumatic stress symptoms.

** *P* < 0.01.

To quantify the strength of the relationship between the variables, we calculated Pearson correlation coefficients (see Table 1). Making and receiving offers of help on social media were positively associated with each other, indicating that people who received help were more likely to give help and viceversa. Both receiving and making offers of help on social media were positively associated with PTG, but there was no significant correlation with PTS. A positive association was found between PTG and PTS at W2.

Two separate sets of linear regression analyses were performed to investigate whether (i) making and/or (ii) receiving offers of help by means of social media (W1) predicted subsequent PTS and PTG (W2), above and beyond background variables (see Table 2). Both regressions were controlled for W1 covariates in Step 1 (age, gender, marital status, education, and exposure to the fire) when predicting W2 outcomes. In addition, for the PTS outcome, we controlled for PTG, while for the PTG outcome we controlled for PTS. We then entered both making and receiving offers help by means of social media at W1, in the second step. Results showed that receiving offers of help on social media predicted higher PTG (*β* = 0.28; *t* = 4.13; *P* < 0.00) as did offering help by means of social media (*β* = 2.71; *t* = 3.97; *P* < 0.00). The final model explained 26.3% of the variance of PTG. However, no significant association was found between offering help by means of social media and PTS levels at W2, nor receiving offers of help by means of social media and PTS at W2, and the addition of these variables did not explain any additional variance beyond the first step, which explained 27.2% of the variance in PTS.

**TABLE 2:** Linear Regression for Variables Predicting PTG and PTS (N = 212)

		PTG			PTS		
		B	SE	β	B	SE	β
Step1	(Constant)	6.97	3.107		21.62	5.38	
	Age	−.037	.042	−.075	−.076	.075	−.075
	Gender	−1.88	1.206	−.110	−3.111	2.151	−.091
	Relationship status	1.376	1.086	.118	−6.125	1.894	−.235**
	Education	−.579	.706	−.059	−.433	1.259	−.022
	Previous exposure	.152	.211	.055	2.064	.345	.376***
	Distance from fire	−.431	.268	−.109	.83	.481	.011
	PTS	1.66	.039	.331***	−	−	−
	PTG	−	−	−	.526	.124	.263***
R^2^	.166				.272		
Step 2	(Constant)	.926	3.273		21.077	6.011	
	Age	−.056	.040	−.112	−.068	.076	−.068
	Gender	−1.164	1.147	−.068	−3.161	2.168	−.092
	Relationship status	1.623	1.025	.124	−6.187	1.903	−.236**
	Education	−.833	.669	−.084	−.348	1.272	−.081
	Previous exposure	.120	.201	.044	2.100	.349	.383***
	Distance from fire	.282	.259	.071	.137	.493	.017
	PTS	.150	.037	.300***			
	PTG				.539	.133	.270***
	Making offers of help on social media	1.387	.459	.208***	−.667	.889	−.050
	Receiving offers of help on social media	.492	.185	.185***	.154	.357	.029
R^2^	.263				.272		

Abbreviations: PTG, posttraumatic growth; PTS, posttraumatic stress symptoms.

** *P* < 0.01.

*** *P* < 0.001.

## DISCUSSION

To the best of our knowledge, the present study is the first to examine the relationship between offering and/or receiving offers of help by means of social media following exposure to traumatic events with both PTS and PTG. Our results show that, while both offering and receiving more offers of help by means of social media were related to higher subsequent PTG at W2, there was no association with PTS.

Two theories bring to light the potential positive impact of active social media use following trauma exposure. Using the “Uncertainty Reduction” theory^43^ as a lens, researchers who have studied postdisaster media use have argued that information-seeking is a common response among individuals, and serves as a means for individuals to alleviate anxiety induced by the resulting uncertainty of the disaster context.^44^ This tendency is particularly relevant in threatening situations and invites reappraisal of uncertainty feelings by means of active information seeking.^45^ While in the past, individuals turned to traditional media sources for information, social media is increasingly serving this purpose.

An additional theory, the “Uses and Gratifications” theory (UGT) is an approach to understanding why and how people actively seek out specific media to satisfy specific needs.^46^ UGT discusses how users deliberately choose media that will satisfy certain needs and allow individuals to enhance knowledge, relaxation, social interactions, diversion, or escape.^47^ According to these two theories in the context of social media use following traumatic events it is reasonable to assume that people with more PTS are more likely to actively seek out more social media information and interactions relating to the event and at the same time it suggests a possible beneficial outcome of this social media use. However, these theories explain the potential contribution of social media use, but do not explain the need for making and receiving offers of help.

Previous research showed that exposure to emotional responses on social media was, on one hand, associated with psychological distress,^48^ and on the other hand, there is evidence showing that emotional contagion of positive emotions can spread through online social networks.^49^ Additionally, social media might have a positive effect on framing of the traumatic event as has been showed with traditional media.^50^ Our results indicated that making and receiving offers of help on social media may enhance PTG; however, PTG and PTS often coexist,^51^ and we found a lack of association between social media use of both making and receiving help and PTS. This supports a possible explanation found in previous studies showing that PTS and PTG had different predictive paths.^52,53^ Social media use of offering and receiving help predicted PTG but not PTS. This finding might also be explained by previous study that found an association between PTSD and media exposure only when the media source was perceived as stressful.^54^

Our results show that offering help and receiving offers of help are connected to each other and both might be psychologically beneficial. Social media provides an immediate and potentially constant source of social support. The benefits of receiving support from others are well established; however, supporting others is also beneficial and leads to increases in sense of belonging,^55^ increased self-esteem,^56^ self-worth,^57^ social connection,^58^ and sense of control.^59^ These findings add to recent studies that point to a reward-related psychological mechanisms of giving support.^60^

Earlier research suggested that PTG is facilitated when positive cognitions are translated into positive action.^61,62^ Offering help through any kind of medium, including on social media, could be described as an action-focused coping mechanism that might explain the association between offering help and PTG. Tandoc and Takahashi^63^ found that social media provided a means for trauma survivors to interact and participate in the social construction of their experience. In addition, Hall et al.^23^ found that participants who reported higher psychological distress actively participated in social media, which may potentially influence their interpretation of the event and improve resiliency.

A literature review on coping with trauma^64^ indicated that the cultural context of the participants should be taken into consideration to understand the cognitive, behavioral, and emotional processes of individuals following exposure to traumatic events. Therefore, it is possible that, in more collectivistic societies such as Israel, the action of offering and asking for help might turn one’s individual coping to a collective coping. Furthermore, online social contact is connected to lower feelings of loneliness,^65^ and perhaps most importantly in creating a sense of community.^66^ Our results support the findings of Reifegerste et al.^67^ that social support in online communities depends on an individual’s activity, and that online activity is significantly correlated with perceived informational and emotional support.

The results of this study contribute to a growing body of literature suggesting that support giving is a protective factor and can benefit mental health. However, some limitations require attention. First, self-report questionnaires do not represent objective information on social media use, both in terms of intensity of use, and in terms of content. Second, the participants reported on their social media use between 2 and 4 wk after being exposed to the fire, so their responses may be subject to recall bias. Third, the study is based on a convenience sample which limits generalization of the findings. Future research is needed to examine the long-term interactions of objective active social media use following trauma exposure and both positive and pathological mental health outcomes.

## CONCLUSIONS

This is one of the first studies to highlight empirically the advantages of social media in the aftermath of trauma exposure. The current study has clinical implications, indicating that both receiving and offering help could promote positive adaptation and growth, although might not reduce psychological distress nor mitigate posttraumatic symptoms. Connecting with online social networks that promote a viable source of social support and sense of community, may be helpful for individuals following trauma exposure. Social media use is increasing exponentially; understanding the potential role it could have in recovery from trauma exposure, particularly when whole communities are affected, is crucial, and many more studies are needed in this vein.
